# Integrative analysis of breast cancer reveals prognostic haematopoietic activity and patient-specific immune response profiles

**DOI:** 10.1038/ncomms10248

**Published:** 2016-01-04

**Authors:** Frederick S. Varn, Erik H. Andrews, David W. Mullins, Chao Cheng

**Affiliations:** 1 Department of Genetics, Geisel School of Medicine at Dartmouth, Hanover, New Hampshire 03755, USA; 2 Department of Microbiology and Immunology, Geisel School of Medicine at Dartmouth, Lebanon, New Hampshire 03766, USA; 3 Norris Cotton Cancer Center, Geisel School of Medicine at Dartmouth, Lebanon, New Hampshire 03766, USA; 4 Institute for Quantitative Biomedical Sciences, Geisel School of Medicine at Dartmouth, Lebanon, New Hampshire 03766, USA

## Abstract

Transcriptional programmes active in haematopoietic cells enable a variety of functions including dedifferentiation, innate immunity and adaptive immunity. Understanding how these programmes function in the context of cancer can provide valuable insights into host immune response, cancer severity and potential therapy response. Here we present a method that uses the transcriptomes of over 200 murine haematopoietic cells, to infer the lineage-specific haematopoietic activity present in human breast tumours. Correlating this activity with patient survival and tumour purity reveals that the transcriptional programmes of many cell types influence patient prognosis and are found in environments of high lymphocytic infiltration. Collectively, these results allow for a detailed and personalized assessment of the patient immune response to a tumour. When combined with routinely collected patient biopsy genomic data, this method can enable a richer understanding of the complex interplay between the host immune system and cancer.

## Abstract (web summary)

Tumour samples are heterogeneous and are comprised of multiple cell types in addition to cancer cells. Here, the authors devised a method to estimate the relative levels of haematopoietic cells in breast cancer samples and demonstrate that this correlates with prognosis.

## Graphical abstract

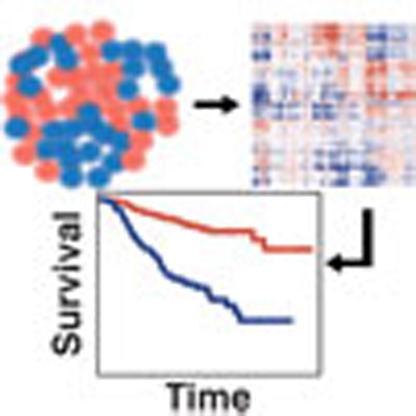

Solid tumours are highly heterogeneous and are made up of cancer cells and a variety of haematopoietic cells that comprise the tumour's microenvironment^1^. Understanding how these cell subsets co-evolve *in situ* can allow for improved characterization of patient tumours, while providing valuable information with regard to prognostic prediction and therapeutic response. Gene expression profiling has traditionally been used to understand the role of neoplastic gene expression programmes in tumour growth and progression. However, gene expression activity in tumour-associated haematopoietic cells can alter the neoplastic gene expression profile, making it possible to infer a tumour's cellular composition. In recent times, many methods have taken advantage of these alterations to computationally estimate immune activity in biopsies from solid tumours^2^,^3^,^4^,^5^. These approaches have yielded interesting results, inferring the presence of haematopoietic subpopulations in several cancers using the tumour's expression of lineage-defining signature genes. However, infiltrating haematopoietic cells affect the composite tumour expression profile transcriptomically, providing additional information that is not captured by signature-based methods. Accounting for this information can improve the sensitivity of these analyses, leading to a more accurate portrayal of the tumour microenvironment.

The Immunological Genome Project is a joint effort between immunologists and computational biologists to transcriptomically profile the murine immune system using carefully controlled methods of sample collection and data analysis^6^. To date, over 200 haematopoietic lineages have been profiled, making this one of the most comprehensive gene expression data sets related to haematopoiesis. The high conservation between murine and human immune profiles makes this data set a rich resource for probing human haematopoietic subpopulations found in patient tumours^7^. Here we use this data set to compare the relative activity of different haematopoietic expression programmes between patient tumours. Using breast cancer as our model, we demonstrate that activity from several lineages correlates with patient survival, and that many of these programmes are associated with the presence of infiltrating haematopoietic cells. We provide functional context to our results by investigating each lineage's association with immune-related gene expression and analysing the role of each haematopoietic lineage across breast cancer subtypes. In addition, we validate our method by applying it to additional cancer data sets and comparing our results obtained using murine haematopoietic profiles with those from human profiles. Together, these results allow us to sensitively characterize the haematopoietic activity of the tumour microenvironment and predict both anticipated and unanticipated cell combinations that are prognostically significant for patient care.

## Results

### Survival analysis of haematopoietic activity in breast cancer

A schematic of our analysis is shown in Supplementary Fig. 1. The BASE algorithm^8^ was implemented to quantify the rank similarity between a breast cancer patient's gene expression profile and each of the 230 murine haematopoietic lineage profiles from the Immunological Genome Project^6^. When iteratively applied to the 1,992 patients from the METABRIC data set by Curtis *et al.*^9^, this resulted in a 1,992 × 230 matrix of rank-similarity metrics, hereby referred to as Cell Lineage Scores (CLSs). Each patient's CLS indicates the relative activity of a cell type compared with that of other patients. As such, CLSs are useful in distinguishing differing haematopoietic activity between patients. Each lineage's CLSs were used as continuous variables in a univariate Cox proportional hazards (PH) model, to identify haematopoietic lineages that were prognostically significant (Supplementary Data 1). We evaluated the sensitivity of this analysis by replacing the haematopoietic profiles in our workflow with a randomized haematopoietic data set that was generated by permuting each gene's expression levels across haematopoietic cell types. The resulting CLSs from the permuted data were not significantly correlated with patient survival.

Haematopoietic lineages are diverse, consisting of stem cells, innate and adaptive immune cells and stromal cells. To account for these differences in function, the haematopoietic lineages were grouped into three categories: dedifferentiated, innate and adaptive. The dedifferentiated category included stem cells and developmental B cells found in the bone marrow and fetal liver, as well as developmental T cells found in the thymus. The innate category included cells involved in innate immunity, specifically dendritic cells (DC), macrophages, CD11b+ myeloid cells, monocytes, natural killer (NK) cells and plasmacytoid DCs (pDC). Finally, the adaptive category encompassed the cells involved in adaptive immunity including B cells, CD4+ memory/naive T cells, CD8+ effector/memory/naive T cells, γδ T cells, natural killer T (NKT) cells, regulatory T cells and T-helper (Th) cells.

**Figure 1:**
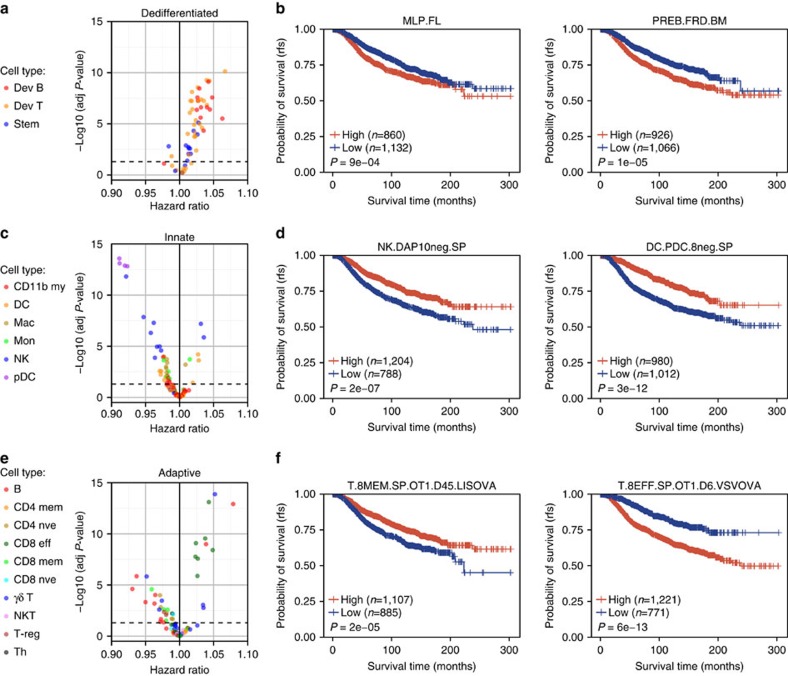
Haematopoietic lineage survival associations. (**a**) Distribution of the HRs and adjusted *P*-values (Wald's test) derived from univariate Cox PH models that included dedifferentiated murine haematopoietic cell type CLSs as the variables. Dedifferentiated cell types include developmental B and T cells (Dev B and Dev T, respectively), and stem cells. Each point corresponds to a different lineage and each colour represents a different haematopoietic subtype. Cell types above the dotted line are associated with survival at an adjusted *P*-value <0.05. (**b**) Kaplan–Meier plot depicting the survival probability over time for samples with high (red) and low (blue) CLSs for two example dedifferentiated cell types, the multi-lineage progenitor from fetal liver and Fr.D pre-B cell. (**c**) Volcano plot displaying univariate Cox PH model results using innate immune lineage CLSs as variables. Innate immune cell subtypes consist of CD11b+ myeloid (CD11b my), DC, macrophages (Mac), monocytes (Mon), NK and pDC. (**d**) Kaplan–Meier plot depicting survival probability over time for two example innate immune cell types, the DAP10− splenic NK cells and CD8− pDCs. (**e**) Volcano plot displaying results from univariate Cox PH models that used adaptive immune lineage CLSs as variables. Adaptive immune cell subtypes include B, CD4+ memory T (CD4 mem), CD4+ naive T (CD4 nve), CD8+ effector T (CD8 eff), CD8+ memory T (CD8 mem), CD8+ naive T (CD8 nve), γδ T, NKT, regulatory T (T-reg) and Th cells. (**f**) Kaplan–Meier plot depicting the survival probability over time for an example CD8+ memory T cell and CD8+ effector T cell. For all Kaplan–Meier plots, samples were stratified into high and low groups based on whether their CLS was above or below the modal frequency of the CLS distribution for the given cell type. *P*-values were calculated using the log-rank test. Vertical hash marks indicate censored data.

CLSs from 75% of the dedifferentiated profiles were associated with poor survival (hazard ratio (HR)>1, adjusted *P*<0.05; Fig. 1a). This effect is demonstrated in patients dichotomized into high and low CLS groups for the multi-lineage progenitor derived from fetal liver and the Fr.D pre-B cell (Fig. 1b). CLSs for 39% of the innate profiles were significantly associated with good prognosis (HR<1, adjusted *P*<0.05; Fig. 1c). Specifically, high CLSs for pDCs, NKs and macrophages were indicators of good survival, whereas associations for DCs, CD11b+ myeloid cells and monocytes were mixed. This relationship can be further examined in the survival distributions for samples dichotomized by CLS for DAP10− splenic NK cells and CD8− pDCs (Fig. 1d). Survival association with adaptive immune CLSs was cell-type dependent. Protective lineages included a variety of B cells, CD4+ naive T cells and CD8+ memory T cells, whereas the deleterious lineages were primarily CD8+ effector T cells (Fig. 1e). The opposing survival distributions for samples dichotomized by CLS for example CD8+ memory and effector T cells are shown in Fig. 1f. Overall, the results from the univariate Cox PH model were comparable to those using multiple Cox PH regression adjusting for common clinical variables including age at diagnosis, stage, oestrogen receptor status, HER2 status and progesterone receptor status (Supplementary Data 2). Taken together, these results depict human breast tumours as highly heterogeneous, with numerous haematopoietic cell-related programmes seemingly involved in patient survival.

We compared our results obtained using full haematopoietic expression profiles with those obtained using minimal gene sets we defined to represent each lineage (Supplementary Data 3 and Methods). Of the 132 lineages whose full expression profiles were significantly associated with patient survival in the univariate analysis, 63 had significantly prognostic gene sets (Supplementary Data 4). These lineages were primarily developmental B cells, developmental T cells, stem cells, CD8+ effector T cells and NK cells. This result suggested that certain lineages survival contribution could be recapitulated using a small number of genes; however, the low overall number of consistent gene sets underscored the importance of using the entire gene expression profile when identifying survival-associated haematopoietic programmes.

### Associations with tumour purity

**Figure 2:**
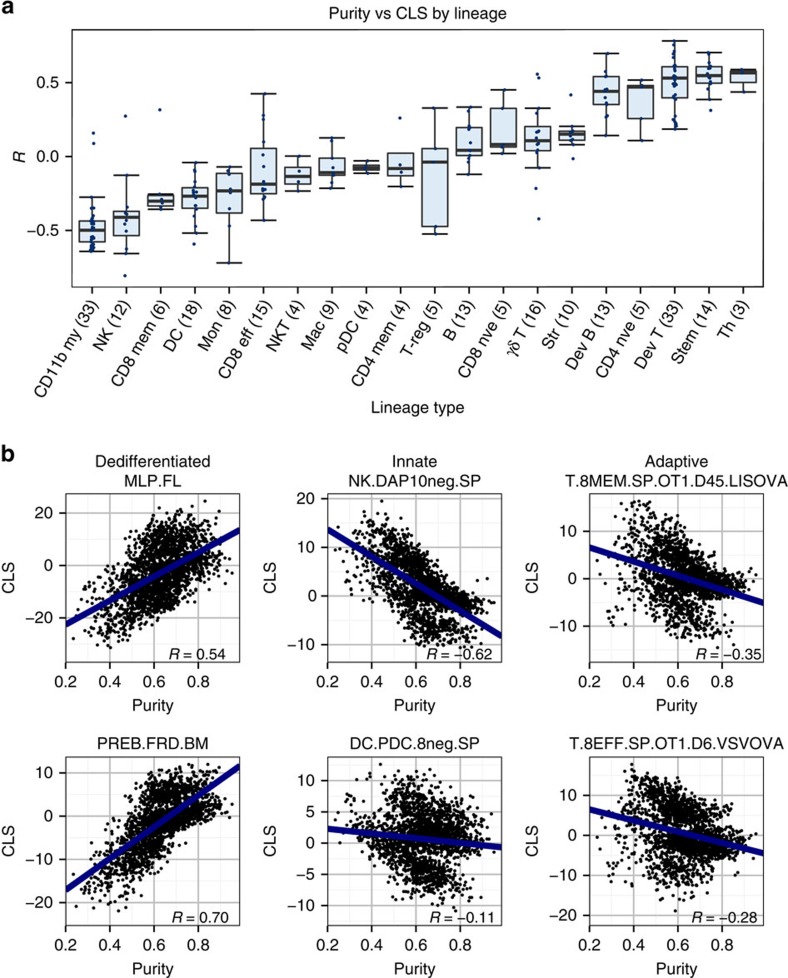
Correlations between haematopoietic lineage CLS and ESTIMATE purity score. (**a**) Box plot depicting each haematopoietic subtype's distribution of Pearson's correlation coefficients for purity–CLS correlations. Cell subtypes include CD11b+ myeloid (CD11b my), NK, CD8+ memory T (CD8 mem), DC, monocytes (Mon), CD8+ effector T (CD8 eff), NKT, macrophages (Mac), pDC, CD4+ memory T (CD4 mem), regulatory T (T-reg), B, CD8+ naive T (CD8 nve), γδ T, stromal (Str), developmental B (Dev B), CD4+ naive T (CD4 nve), developmental T (Dev T), stem and Th cells. The numbers in parentheses indicate the number of cells of each lineage type in the Immunological Genome Project data set. Each box spans quartiles with the lines representing the median correlation coefficient for each group. Whiskers represent absolute range excluding outiers. All outliers were included in the plot. (**b**) Scatterplots representing the association between ESTIMATE tumour purity score and CLS for two cell types each from the dedifferentiated, innate and adaptive haematopoietic categories.

Tumour biopsies typically comprise a variety of cell types from the tumour microenvironment, including stromal and infiltrating immune cells, in addition to the tumour cells. To stratify infiltrate-related transcriptional programmes from tumour-related ones, we correlated patient CLSs for each lineage with tumour purity scores calculated using the ESTIMATE algorithm^2^ (Fig. 2a). Briefly, ESTIMATE uses single-sample gene set enrichment analysis of a stromal and immune signature to generate scores that represent the presence of these cell types in tumour tissue. ESTIMATE then combines these scores to infer tumour purity. The resulting correlations revealed that CLSs from dedifferentiated cell types such as the multi-lineage progenitor and Fr.D pre-B cell were strongly associated with tumour purity (*R*=0.54 and 0.70, respectively), suggesting that dedifferentiated programmes are intrinsic to the tumour itself (Fig. 2b). Contrarily, adaptive and innate immune CLSs tended to be negatively associated with tumour purity, indicating that these CLSs are capturing immune infiltrate-related gene expression. To highlight, two cell types from the innate category, DAP10− splenic NK cells and CD8− pDCs, had Pearson's correlation coefficients of −0.62 and −0.11, respectively, whereas two example adaptive lineages, CD8+ memory and effector T cells, had coefficients of −0.35 and −0.28, respectively (Fig. 2b). Interestingly, CLSs from CD4+ naive and Th cells had purity correlations consistent with dedifferentiated expression programmes. However, a whole-genome expression correlation between these cell types and dedifferentiated cell types revealed that CD4+ naive and Th cells were transcriptionally similar to the developmental T cells (Supplementary Data 5). Thus, these correlations were probably a function of tumour-related gene expression programmes the CLS was capturing.

As the ESTIMATE algorithm calculates purity scores using the same gene expression information used to determine the CLS, we validated our purity correlations using an orthogonal measure of tumour purity that inferred lymphocytic infiltration (LI) based on image analysis of stained tissue slides^10^. This method had previously been applied to the METABRIC data set to calculate LI scores for 564 samples. We used these scores to stratify samples about the median into high and low LI groups and then examined the difference between each group's CLSs for each lymphocytic lineage (Supplementary Fig. 2). Our analysis revealed that the LI-high group had significantly higher CLSs in 60 of the 88 lymphocytic lineages we tested (*P*<0.05, two-sided *t*-test), suggesting that lymphocytic CLSs are primarily capturing gene expression programmes associated with infiltrating immune cells.

### Spatiotemporal patterns of haematopoietic activity

**Figure 3:**
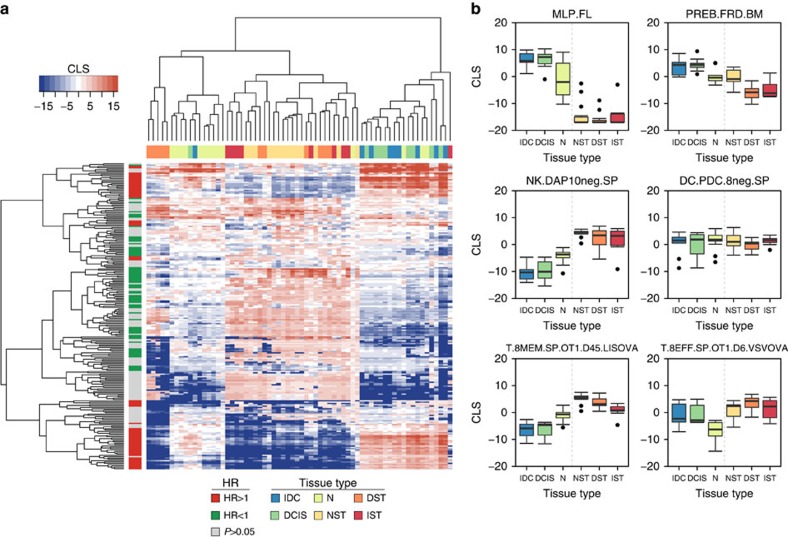
Spatiotemporal arrangement of haematopoietic lineages. (**a**) Heatmap depicting the hierarchical clustering of CLSs for each haematopoietic cell type in epithelial and stromal tissue from normal (N, *n*=14; NST, *n*=14), DCIS (*n*=9; DST, *n*=11) and invasive ductal carcinoma (IDC, *n*=9; IST, *n*=9) samples. To show contrast, the max and min CLS were set to the 99th percentile of the original CLS distribution. Row sidebar represents the HRs for each haematopoietic cell type based on the univariate Cox PH survival analysis done in the Curtis data set, with grey indicating adjusted *P*>0.05 (Wald's test), green indicating HR<1 and red indicating HR>1. Column sidebar represents the type of sample. Colours in the column sidebar correspond to the colours in the box plots of **b**. (**b**) Example CLS distributions for each sample tissue type using the data displayed by the heatmap. Two cell types each were chosen to represent the dedifferentiated, innate and adaptive haematopoietic categories. Each box spans quartiles with lines representing the median correlation coefficient for each group. Whiskers represent absolute range excluding outliers. All outliers were included in the plot.

Understanding the spatiotemporal arrangements of immune activity can provide insights into how the immune system and tumour coevolve. We investigated this relationship by examining the distribution of each lineage's CLSs across stromal and epithelial cells derived from normal, ductal carcinoma *in situ* (DCIS) and invasive ductal carcinoma (IDC) tissue using a data set generated by Ma *et al.*^11^ Hierarchical clustering on the basis of CLS revealed distinct clustering patterns, with DCIS and IDC epithelial samples clustering apart from stromal samples and normal epithelial samples (Fig. 3a). CLSs from poor survival lineages, primarily from the dedifferentiated subset, tended to be higher in DCIS and IDC epithelial samples, whereas protective lineages, including most innate and adaptive immune cells, had higher CLSs in stromal samples (sidebar, Fig. 3a). Interestingly, CLS clustering revealed few differences between DCIS and IDC samples, suggesting that dedifferentiation and immune activity play minimal roles in initiating tumour invasion. Similarly, clustering of normal, DCIS and IDC stromal samples revealed few distinct CLS patterns, indicating that high immune activity is a trait of stromal tissue, regardless of neoplastic status. The CLS distributions for select lineages can be seen in more detail in Fig. 3b. Together, these results indicate that tumour immune activity is highest in the stromal region, whereas tumour epithelial cells tend to exhibit dedifferentiation programmes. Although several groups have associated infiltrating immune cells with an invasive phenotype in cancer^12^, our data suggest that immune activity in the stromal compartment is not sufficient to induce an invasive programme and, by extension, that qualitative differences in immune response or multivariate factors in addition to immune activity may be required to mediate invasion.

### Immunological associations with genetic markers

**Figure 4:**
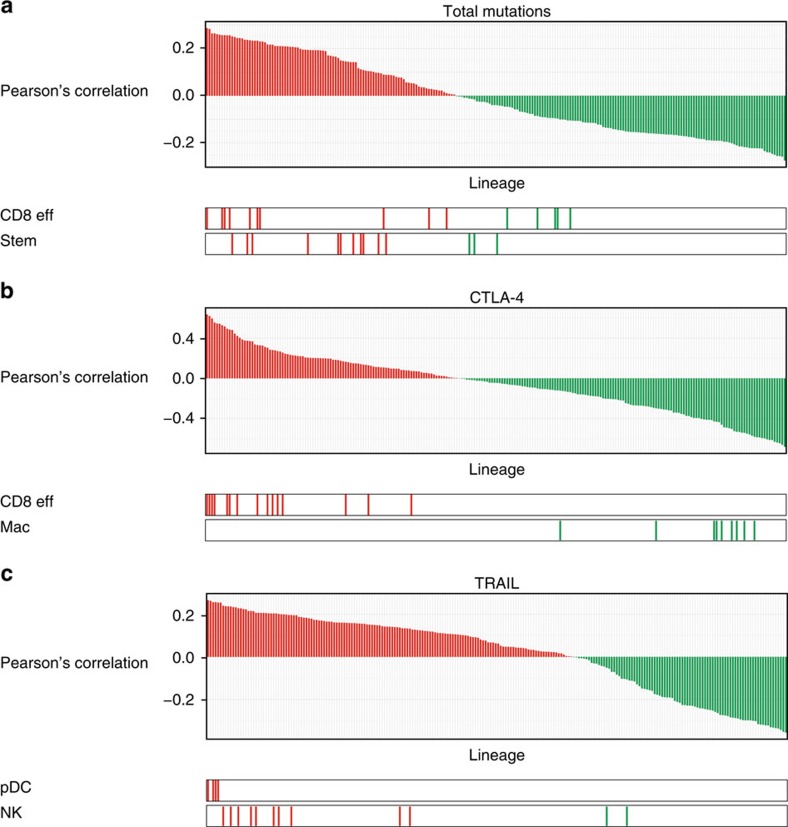
CLS correlations with mutational burden and gene expression. The figure represents ranked Pearson's correlation coefficients describing the association between a sample's CLS for a given cell type and their (**a**) total number of mutations, (**b**) CTLA-4 expression and (**c**) TRAIL expression. In all figures, each bar represents a different cell type, with red and green bars representing positive and negative correlations, respectively. Below each figure, the locations in the rankings of select haematopoietic subtypes are highlighted. These subtypes include CD8+ effector T cells (CD8 eff), stem cells, macrophages (Mac), pDCs and NK cells.

The presence of infiltrating CD8+ effector T cells at a tumour has traditionally been associated with good prognosis in a variety of cancer types^13^,^14^,^15^,^16^,^17^. However, our survival analyses indicated that increased CD8+ effector T-cell activity was associated with poor patient survival. We hypothesized that this may be a function of cancer severity, with higher CD8+ effector T-cell activity found at more severe tumours, in apparent contradiction with the prevailing impression of the field^18^. To test this hypothesis, we used The Cancer Genome Atlas (TCGA) breast cancer data to examine the relationship between the total number of mutations in a sample and its CLSs for each lineage. Correlation analyses revealed that stem cells and CD8+ effector T cells were the cell types most strongly associated with mutational burden, with Pearson's correlation coefficients from both cell types being significantly higher relative to background (*P*=4e−3 and 1e−3, respectively, Wilcoxon rank-sum test; Fig. 4a). The stem cell relationship was not surprising, as high mutational load and dedifferentiation transcription programmes are both markers of severe cancer^19^,^20^,^21^. However, the similar relationship in CD8+ effector T cells was interesting, as it suggested these cells had greater enrichment in severe tumours relative to other immune lineages.

We followed up this analysis by examining the relationship between the CLSs from each lineage and CTLA-4 expression in the Curtis data set. CTLA-4 is a cell surface protein primarily expressed in T cells that binds B7 molecules with high affinity, resulting in T-cell inhibition^22^,^23^,^24^. In the tumour microenvironment, this can limit T-cell activation and function. When the lineages were ranked by their Pearson's correlation coefficients, CD8+ effector T cells, CD4+ memory cells and CD8+ memory cells were significantly enriched at the top of the rankings relative to other lineages (*P*=6e−8, 5e−3 and 2e−3, respectively, Wilcoxon rank-sum test). Interestingly, B7-expressing macrophage cells were significantly enriched at the bottom of the rankings (*P*=1e−4, Wilcoxon rank-sum test; Fig. 4b), indicating a decrease in CTLA-4 levels and perhaps T-cell abundance in their presence. Together, these results suggest that high immunosuppressive activity may be limiting T-cell effector functions in the tumour microenvironment.

Tumour necrosis factor-related apoptosis inducing ligand (TRAIL, CD253) is a cytokine known to induce apoptosis in tumour cells and has commonly been used as a target in anticancer therapies^25^,^26^. Various cells of the innate immune system are known to express TRAIL^27^. To investigate the relationship between immune infiltrate and TRAIL expression, we ranked each lineage based on its Pearson's correlation coefficient between CLS and TRAIL expression (Fig. 4c). Interestingly, we found that pDCs and NK cells were significantly enriched at the top of these rankings (*P*=6e−4 and 9e−4, respectively, Wilcoxon rank-sum test), suggesting that these cells may mediate TRAIL expression as part of their protective effect.

### Patterns of activity across breast cancer subtypes

**Figure 5:**
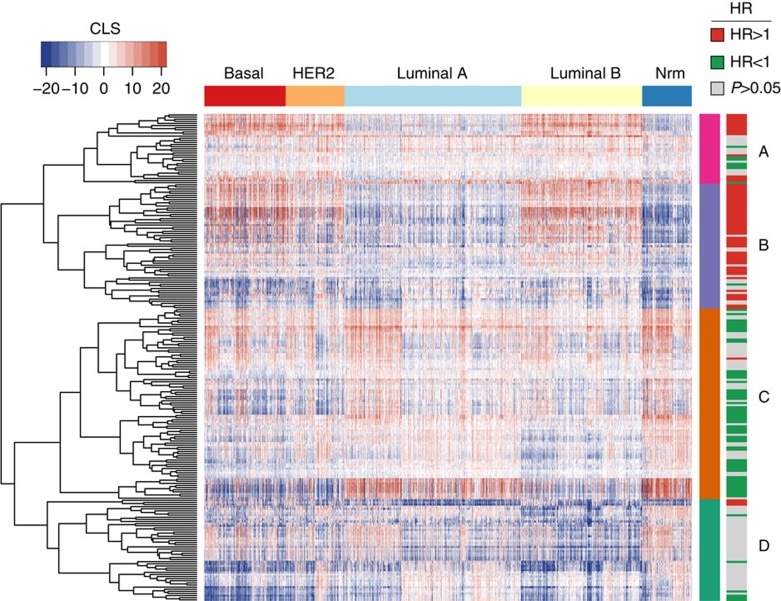
Haematopoietic lineage association with PAM50 subtypes. Heatmap depicting the hierarchical clustering of CLSs for each haematopoietic cell type in basal (*n*=331), HER2 (*n*=240), luminal A (*n*=721), luminal B (*n*=492) and normal-like (Nrm) (*n*=202) tumours (column sidebar). To show contrast, the max and min CLS were set to the 99th percentile of the original CLS distribution. Left row sidebar depicts the four distinct CLS clusters (A–D) that emerged. Right row sidebar represents the HRs for each haematopoietic cell type based on the univariate Cox PH survival analysis done in the Curtis data set, with grey indicating adjusted *P*>0.05 (Wald's test), green indicating HR<1 and red indicating HR>1.

Breast cancer subtyping is useful for characterizing breast cancer samples and predicting prognosis. The METABRIC data set included sample information for both prediction analysis of microarray 50 (PAM50) subtypes^28^,^29^ and the integrative clustering subtypes by Curtis *et al.*^9^ We hypothesized that these subtypes may reflect varying haematopoietic activity encapsulated by each sample's set of CLSs. To examine this association, we first stratified samples by their PAM50 subtype and hierarchically clustered the lineages by CLS across the Curtis data set. Interestingly, this method revealed that four major clusters of 33, 59, 90 and 48 lineages, labelled A–D, respectively, had distinct activity in the individual PAM50 subtypes (Fig. 5). Repeating this method using the Curtis *et al.* subtypes revealed a similar pattern (Supplementary Fig. 3). These four clusters had unique compositions of cell types linked by their prognostic associations, each with distinct activity in the individual subtypes. Cluster A was enriched in adaptive immune cells (*P*=3.2e−6, Fisher's exact test) with varying survival associations. The deleterious cell types in this cluster exhibited high activity in the basal-like, HER2 and luminal B subtypes, whereas the protective lineages did not display clear subtype-specific patterns. Cluster B was enriched in dedifferentiated cell types (*P*=3.9e−25, Fisher's exact test). These lineages tended to be associated with poor survival and had their highest activity in the basal-like, HER2 and luminal B subtypes. Cluster C was characterized by an abundance of innate immune cell types (*P*=4.3e−14, Fisher's exact test). In addition to innate cell types, this cluster also included all the stromal cell types and some adaptive immune cell types. These cell types were primarily associated with good survival and had their highest scores in the luminal A and normal-like subtypes. Finally, cluster D was enriched in haematopoietic lineages with no prognostic association (*P*=1.8e−9, Fisher's exact test). The activity of cells in this cluster showed no distinct patterns across the subtypes. These results indicated that although subtypes are defined by distinct clinical characteristics, they sometimes share similar haematopoietic features.

These relationships were further demonstrated by examining the distribution of PAM50 subtypes after clustering samples by their CLSs (Supplementary Fig. 4). This analysis yielded four distinct clusters of samples, defined by a mixture of either basal-like, HER2 and luminal B subtypes or luminal A and normal-like subtypes. Interestingly, each cluster exhibited a significantly different survival distribution from the others (*P*=3e−6, log-rank test). Taken together, these findings suggest that although the PAM50 subtyping system is capturing the molecular underpinnings of a patient's disease, a novel subtyping system may better reflect the haematopoietic activity present in a patient's tumour.

The differences in haematopoietic activity across subtypes led us to stratify samples by both PAM50 and Curtis *et al.* subtype, and re-examine the association between survival and CLS for each haematopoietic lineage (Supplementary Data 6 and 7). Many of these survival associations were no longer significant after stratification, indicating that subtyping captured much of the haematopoietic diversity within breast cancer samples. However, some lineages remained prognostic in certain subtypes. To demonstrate this finding in more detail, we performed example two class comparisons for lineages that remained significantly predictive of patient survival in individual PAM50 and Curtis *et al.* subtypes (Supplementary Figs 5 and 6).

### Reproducibility of haematopoietic survival analyses

**Figure 6:**
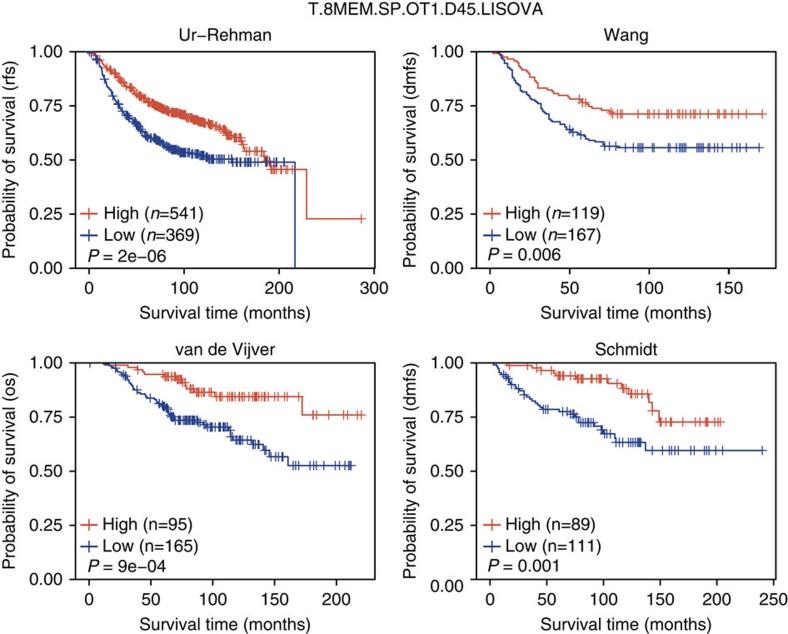
Reproducibility of T8.MEM.SP.OT1.D45.LISOVA CLS survival association in breast cancer data sets. Kaplan–Meier plots depicting the survival probability over time for samples with a high (red) and low (blue) CLS for the T8.MEM.SP.OT1.D45.LISOVA cell type, an example CD8+ memory T cell. Samples were stratified into high and low groups based on whether their CLS was above or below the modal frequency of the CLS distribution for the given cell type. *P*-values were calculated using the log-rank test. Vertical hash marks indicate censored data.

To confirm that our findings were not localized to the Curtis data set, we extended our univariate survival analysis to four additional breast cancer data sets by Ur-Rehman *et al.*^30^, Wang *et al.*^31^, van de Vijver *et al.*^32^ and Schmidt *et al.*^33^ At an adjusted *P*-value of 0.05, 131 lineages were significantly predictive of survival in at least 3 data sets and 69 were significantly predictive across all 5 data sets tested (Supplementary Data 8). These results indicate that the CLSs from a large number of haematopoietic lineages are reproducible measurements of patient survival in breast cancer. Figure 6 shows the survival distributions of samples from each data set stratified by CLSs from an example CD8+ memory T-cell lineage. This lineage was significantly associated with patient survival in all five data sets tested.

### Survival analyses across multiple tumour types

**Figure 7:**
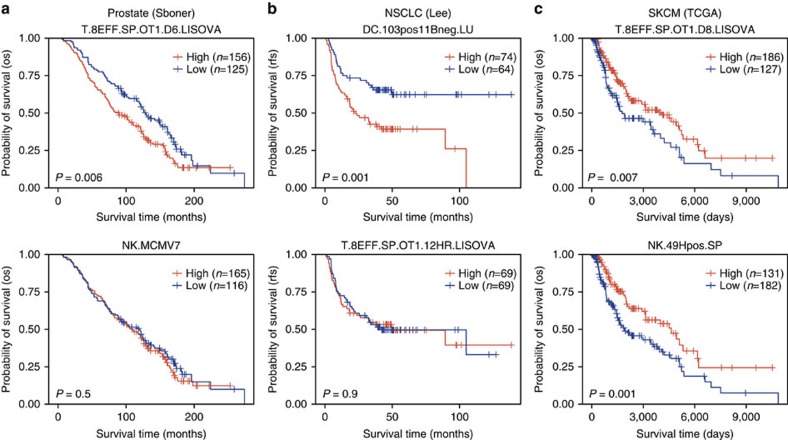
Example CLS survival distributions in three additional cancer types. (**a**) Kaplan–Meier plots depicting the survival probability over time for prostate cancer samples with high (red) and low (blue) CLSs for an example CD8+ effector T cell and activated NK cell. (**b**) Kaplan–Meier plot depicting the survival probability over time in non-small cell lung carcinoma (NSCLC) patients with high and low CLSs for CD103+ CD11b− DCs and an example CD8+ effector T cell. (**c**) Kaplan–Meier plot depicting the survival probability over time in SKCM patients with high and low CLSs for an example CD8+ effector T cell and ly49+ NK cells. For all Kaplan–Meier plots, samples were stratified into high and low groups based on whether their CLS was above or below the modal frequency of the CLS distribution for the given cell type. *P*-values were calculated using the log-rank test. Vertical hash marks indicate censored data.

The strong and robust survival associations our framework identified in breast cancer encouraged us to apply our framework to other tumour types. CLSs were calculated for samples in prostate cancer^34^, non-small cell lung cancer (NSCLC)^35^ and TCGA skin cutaneous melanoma (SKCM) data sets and fitted to univariate Cox PH models (Supplementary Data 9–11, respectively). The results from these survival analyses suggested a cancer-specific role for different immune lineages in patient survival. For instance, in prostate cancer, high CD8+ effector T-cell CLSs were indicative of a poor prognosis and NK cell CLSs were not significantly associated with patient survival (Fig. 7a). In NSCLC, high DC CLSs were associated with poor prognosis, whereas CD8+ effector T-cell CLSs were not significantly associated with patient survival (Fig. 7b). Finally, in SKCM, high CD8+ effector T-cell CLSs and NK-cell CLSs were both associated with prolonged survival (Fig. 7c). Together, these results demonstrate that our method can be applied to numerous cancers and may be a valuable tool going forward in dissecting the role of the immune system in various tumour types.

### Recapitulation of results using human profiles

The associations we identified in this study were determined by assuming that CLSs calculated from murine haematopoietic profiles were representative of analogous underlying human cell populations. To validate this assumption, we used the BASE algorithm to calculate CLSs in two human haematopoietic data sets by Abbas *et al.*^36^ and Novershtern *et al.*^37^ (Supplementary Data 12 and 13). After renormalizing the resulting CLSs across human lineages, this analysis enabled us to globally identify the human haematopoietic lineages that most strongly correspond with the murine lineages. In both data sets, CLSs from the murine lineages were primarily highest in their analogous human lineages, indicating high cell-type similarity between species.

As a follow-up to this analysis, we calculated CLSs in the METABRIC data set using the human haematopoietic gene expression profiles in lieu of the murine profiles. We then fit the resulting CLSs to a univariate Cox PH model and compared the results we obtained using murine profiles with those from each human data set. Surprisingly, only the human immune lineages in the Abbas *et al.* data set were significantly correlated with patient survival. To check for consistency between the Abbas *et al.* results and the results we obtained using the murine profiles, we correlated the human gene expression profiles with each murine gene expression profile, to identify the murine lineage that was most similar to each human lineage. We then compared the survival results between each human profile and its best matched murine profile (Supplementary Data 14). Our analysis demonstrated that each human lineage was most strongly correlated with an analogous murine lineage. In addition, for each human–murine lineage pair where both lineages were significantly predictive of patient survival (adjusted *P*<0.01, Wald's test), the HRs of each lineage indicated a consistent protective or deleterious effect on survival.

## Discussion

We have developed an integrated approach to computationally identify active haematopoietic transcriptional programmes in a patient's tumour sample based on transcriptional similarity to murine haematopoietic profiles. By using continuous transcriptional profiles from 230 haematopoietic cell lineages, our approach provides high sensitivity in classifying which haematopoietic expression programmes are most prominent in patient tumours. Correlation analyses between CLS and tumour purity indicate that these haematopoietic programmes are likely to be associated with infiltrating immune cells in the patient tumour microenvironment or dedifferentiated transcriptional programmes intrinsic to the tumour itself. This systematic approach can be used for a variety of basic science and clinical applications.

Gene expression analyses provide only a snapshot in time of the overall struggle between cancer and the immune system. Keeping this in mind, the presence of cells at a tumour site may simply serve as prognostic biomarkers, even if they are not directly involved in antitumour response. Our survival analyses revealed that the activity of pDCs, NK cells, macrophages, B cells, CD4+ naive cells and CD8+ memory T cells was associated with enhanced survival. Some of these lineages including pDCs and NK cells are well characterized for their antitumour effects^38^,^39^. However, others such as B cells, CD4+ naive cells and CD8+ memory cells are typically found at the site of a previous successful immune response^33^,^40^,^41^,^42^. This complicates our understanding of why infiltrate of each lineage is associated with a survival response. However, quantification of lineage activity at the tumour site using this method remains valuable for prognostic purposes and these results can be used to generate novel hypotheses for future investigation in preclinical and patient-centred trials. If validated, these observations could change our understanding of cancer immunology and inform future immunotherapy approaches.

Surprisingly, we found that the primary immune-related indicator of poor prognosis was the presence of CD8+ effector T cells in the tumour microenvironment. These findings run contrary to many previous reports^13^,^14^,^15^,^16^,^17^. Our attempts to provide functional context to this result revealed that CD8+ effector T-cell activity was strongly associated with mutational burden and the expression of the immunosuppressive protein CTLA-4. This analysis suggested that the presence of CD8+ effector T cells in a tumour might be an indicator of immunoevasive breast cancer. We suspect, based on our results, that CD8+ effector T cells arrive early in the breast tumour development. Once there, they successfully elicit an antitumour response, leading to the presence of CD8+ memory T cells and other protective-associated lineages. However, if the tumour has become immunoevasive, such as by inducing immunosuppression through CTLA-4, infiltrating CD8+ effector cells will be ineffective. As the tumour continues to mature, more CD8+ effectors will be recruited to the tumour to little effect, leading to the negative association between CD8+ effector CLS and patient survival. Alternatively, these data could suggest that classic CD8+ effectors are of less importance than a specific subset of CD8+ memory cells, which in the current analysis cannot be distinguished. For example, recent evidence supports prognostic and therapeutic efficacy of CD103+ tissue-resident CD8+ memory cells in ovarian and lung cancer^43^,^44^.

Although we believe many of the protective lineages found in the tumour are indicative of a previous successful immune response, we observed that NK cells and pDCs correlated well with the expression of the antitumour protein TRAIL. TRAIL is constitutively expressed in NK cells but cells that express interferon-α/β such as pDCs, monocytes and other NK cells have been shown to further induce its expression^38^,^39^,^45^. We suspect that the presence of pDCs in a tumour induces TRAIL expression in NK cells, leading to increased apoptosis in tumour cells, thus conferring a survival-protective effect. Further exploration of this relationship may reveal important details in regards to breast cancer immunotherapy and immunosurveillance.

When applied to three additional cancer types, our method suggested that the role of the immune system on patient survival is strikingly context dependent. Some of these associations have been previously reported. For instance, prostate tumours have been shown to escape NK immunosurveillance by shedding the NK cell-activating ligand NKG2D^46^, which is in agreement with our finding that NK cells are not significantly associated with patient survival. In addition, our results relating to the differing roles of CD8+ effector T cells in melanoma and NSCLC patients agree with previous reports that CD8+ effector T cells have a protective role in melanoma patients and no association with survival in NSCLC patients^47^,^48^. However, other associations we found had not been well characterized, such as the negative association between DCs and NSCLC patient survival. Understanding the different avenues by which the immune system influences patient survival across cancer types will have important implications regarding immunotherapy in the future. The systematic manner by which our approach associates haematopoietic programmes with patient survival should enable more detailed analyses going forward.

Our approach employs a user-selected haematopoietic gene expression data set to query the cell-specific programmes active in a patient's tumour. This data set should be carefully chosen to ensure that the resulting associations faithfully represent the underlying biology. Taking this into consideration, we established the use of murine haematopoietic profiles as proxies for measuring human haematopoietic subpopulations. Murine profiles offer several advantages over human profiles, as mice can be easily manipulated to allow for comprehensive genomic profiling and diminished variability between experiments. By using this resource, we were able to detect activity for several haematopoietic lineages whose role in cancer had not been extensively characterized. However, many of the cell types in this data set were highly correlated, diminishing the specificity of our analysis. Going forward, this method could be improved through employing a data set that better contrasts related cell types. Alternatively, the BASE algorithm could be modified to place greater weight on the genes that best define each individual cell type. By applying these changes, a more thorough depiction of the tumour haematopoietic landscape can be obtained.

As demonstrated, using our approach to examine associations between immune activity, patient survival and cytogenetic features can generate numerous hypotheses. Experimental validation of these prospective models will be critical in further understanding the interplay between the immune system and cancer. Going forward, this method can be implemented in consort with sequencing data, to identify potential immunogenic mutations that may alter the cellular makeup of the tumour microenvironment. In addition, as our preliminary results in multiple tumour types demonstrate, this method can be applied in a pan-cancer manner to compare the immune system's role in different disease contexts. The high sensitivity of this method enables multiple follow-up analyses with the potential to provide important insights into tumour immunology. By combining these insights with mechanistic experimentation, the immune system's role in cancer management can be further elucidated.

## Methods

### Gene expression and clinical data

Gene expression and associated clinical data sets were obtained from the European Genome-Phenome Archive (EGAS00000000083), the Gene Expression Omnibus (GSE15907, GSE22886, GSE24759, GSE14548, GSE47561, GSE2034, GSE11121, GSE16560 and GSE8894) and from the website of the Netherlands Cancer Institute (http://ccb.nki.nl/data/). In addition, BRCA and SKCM level 3 gene expression data from the RNAseqV2 platform and the associated clinical data were obtained from the TCGA data portal (http://cancergenome.nih.gov/). Raw gene expression data from the Immunological Genome Project (GSE15907), obtained in July 2014, was background corrected using Robust Microarray Analysis, quantile normalized and fitted with a multichip linear model for each probeset using the ‘expresso' function of the ‘affy' library in R^49^. Probes from the Affymetrix MoGene-1_0-st, HG-U133A, HT_HG-U133A, U133_X3P, HG-U133Plus2.0 and Illumina 6k Transcriptionally Informative Gene Panel for DASL platforms were collapsed into gene symbols using the probe with the highest average intensity across all samples. Murine transcripts from the Affymetrix MoGene-1_0-st platform were matched to human transcripts using gene symbol.

### Pre-processing of haematopoietic profiles

To calculate the CLS using the BASE algorithm^8^, haematopoietic gene expression profiles must first be transformed so that each value reflects the gene's relative expression level in a given cell type. Beginning with a haematopoietic data set (GSE15907, GSE22886 or GSE24759), relative gene expression values are obtained by median normalizing the absolute expression values across cell types. From there, each cell type's relative gene expression values are *z*-transformed so that they follow a standard normal distribution. At this point, *z*-scores >0 mark genes that are upregulated in a given cell type relative to other cells in this data set, whereas *z*-scores <0 mark the genes that are downregulated in a given cell type relative to the other cells. If necessary, replicate gene expression profiles for each cell type are collapsed at this step by taking the mean *z*-score of each replicate. Each newly collapsed cell type's *z*-scores are then *z*-transformed again to renormalize. From there, each cell type's *z*-score profile is split into an up- and downregulated profile. In the upregulated profile, genes with a *z*-score >0 maintain their value while genes with a *z*-score <0 are converted to 0, whereas the downregulated profile maintains the *z*-scores <0, converting *z*-scores >0 to 0. By splitting the profiles into up- and downregulated profiles, *z*-scores can now be converted into *P*-values without losing information regarding the relative expression of a gene in each cell type. The *P*-values are then −log10 transformed, to add more weight to genes that exhibit higher differential expression. To avoid outliers, transformed values >10 are then trimmed to 10. Finally, the transformed values are divided by the maximum value in the data set, to rescale values from 0 to 1.

To create a random data set to test the sensitivity of our method, each gene's expression values from GSE15907 were shuffled between haematopoietic lineages to create novel cell profiles. This shuffled data was then subjected to the pre-processing steps described above. To create minimal gene sets for each lineage in GSE15907, the pre-processed haematopoietic upregulated profiles were filtered to only include genes whose values were equal to 1, indicating strong differential expression, in at least one lineage. This resulted in a simplified matrix containing each lineage's upregulated profile values in 997 genes (Supplementary Data 3). Each lineage had a transformed value of 1 in at least 5 of the 997 genes included in the matrix.

### Calculation of the CLS

The BASE algorithm calculates the CLS by applying the transformed haematopoietic profiles/gene sets to patient gene expression data. The up- and downregulated profiles for each haematopoietic cell type are treated as a weight vector **w**=[*w*_1_, *w*_2_, *w*_3_…*w*_*j*_…*w*_*n*_], where *w*_*j*_=−log_10_(p-val) for gene *j* in that cell lineage and *n*=number of genes. Each patient's gene expression values are log transformed and median normalized across samples, to reflect each sample's relative expression level. The relative expression levels are then ordered from high to low and defined as the vector **g**=[*g*_1_, *g*_2_, *g*_3_…*g*_*j*_…*g*_*n*_], where *g*_*j*_=the relative expression value of gene *j* in a given patient and *n*=number of genes. BASE then inputs these two vectors into a foreground and background function, which are used to calculate a ‘pre-CLS' for the upregulated (*pCLS*_up_) and downregulated (*pCLS*_dn_) profiles:

The foreground function calculates the cumulative distribution of the patient gene expression values weighted by their corresponding transformed hematopoietic expression values:


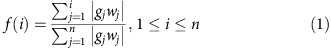


The background function calculates the cumulative distribution of the patient gene expression values weighted by a value complementary (1−*w*) to their corresponding transformed haematopoietic expression values:


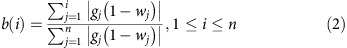


The *pCLS*_up_ and *pCLS*_dn_ are then each calculated by taking the maximum absolute deviation between their respective foreground and background functions. The maximum deviation could be the maximum difference when the foreground function is larger than the background function (*pCLS*^+^) or the minimum difference when the background function is larger than the foreground function (*pCLS*^−^). As a result, these two differences must be compared:


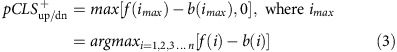



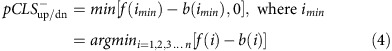



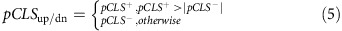


The resulting statistic provides information regarding the similarity between a patient gene expression profile and the up/downregulated haematopoietic profile. For patients whose gene expression profiles closely match an upregulated haematopoietic cell profile the foreground cumulative distribution function will sharply increase early, as highly expressed patient genes in vector **g** are assigned high corresponding haematopoietic weights from vector **w**, before plateauing later due to lowly expressed patient genes being assigned low weights. The background cumulative distribution function will behave conversely, with highly expressed genes assigned low complementary weights and lowly expressed genes assigned high complementary weights. When the maximum deviation between the two functions is calculated, the resulting *pCLS*_up_ will be high, corresponding to the high similarity between the patient and haematopoietic expression profiles. For patient profiles that are compared with concordant downregulated haematopoietic profiles, high weights will correspond to lowly expressed haematopoietic genes. As a result, the roles of the foreground and background cumulative distribution functions will be swapped relative to the upregulated function and the high similarity will result in a highly negative *pCLS*_dn_. For patient expression profiles that are compared with highly discordant haematopoietic profiles, both the foreground and background functions are expected to increase randomly, as the haematopoietic weights will not correspond to high or low expression values. This will result in a low maximum deviation between the two cumulative distribution functions and a low *pCLS*_up/dn_.

To normalize the *pCLS*_up_ and *pCLS*_dn_, the gene labels in vector **g** are permuted 1,000 times to obtain 1,000 permuted gene expression vectors **g**^1^, **g**^2^,…, **g**^1,000^. For each permutation, a *pCLS*_up/dn_ is then recalculated by replacing **g** in equations (1) and (2) with the respective permuted expression vector. This procedure results in 1,000 *pCLS* variables that are used to generate a null *pCLS* distribution. The *pCLS*_up_ and *pCLS*_dn_ are then normalized by dividing each score by the mean of the absolute value of the permuted *pCLS* variables to yield the *CLS*_up_ and *CLS*_dn_. The final CLS is then obtained by subtracting the *CLS*_dn_ from the *CLS*_up_.

### Survival analyses

Survival-associated haematopoietic lineages were identified using a univariate Cox PH model fitted to a given lineage's CLSs across all breast cancer samples. To correct for potential confounders of the univariate analysis in the Curtis *et al.* data set, we performed multiple Cox PH regression, incorporating the CLS, as well as age at diagnosis, stage, oestrogen receptor status, HER2 status and progesterone receptor status. Significance of the model parameters was determined using Wald's test and the resulting *P*-values were adjusted using the Benjamini–Hochberg procedure. Survival distributions were visualized using Kaplan–Meier curves, with samples dichotomized into high- and low-risk groups about the modal frequency of their CLS distribution. Significance of the difference between the survival distributions was assessed using a log-rank test.

Survival analyses were performed in R using the ‘coxph', ‘survfit' and ‘survdiff' functions of the ‘survival' package for Cox PH models, Kaplan–Meier plots and log-rank tests, respectively.

### Haematopoietic lineage grouping

GSE15907, which originally contained 237 haematopoietic lineages, was filtered to remove seven cell types that were either control samples or ambiguously named (CD4posTESTNA, CD4posTESTDB, CD4TESTJS, CD4TESTCJ, CD19CONTROL, CD4CONTROL and AG). The remaining 230 lineages were assigned a group and subgroup based on the type of haematopoietic cell they represented. The developmental B-cell group was defined as all early-phase B cells found in the fetal liver and bone marrow. The developmental T-cell group was defined as all thymal αβ T cells and immature thymal γδ T cells. All CD11b+ myeloid cells were assigned to the CD11b+ myeloid cell category, regardless of myeloid subtype. The remaining subgroups were assigned based on characterization done by the Immunological Genome Project (http://www.immgen.org/)^6^.

### Tumour purity estimation

Gene expression-based tumour purity was estimated in R using the ‘estimate' package^2^. For a given data set, the ‘filterCommonGenes' function was used to select for the genes used by the ESTIMATE algorithm. The ‘estimateScore' function then used the expression levels of these genes to calculate an ESTIMATE score for each sample. From there, these scores were inputted into the previously calculated tumour purity formula to infer a sample's purity. ESTIMATE-based tumour purity values were correlated with CLSs for each haematopoietic lineage using the ‘cor' function in R. LI scores calculated from eosin-stained tissue sections of 564 METABRIC samples were downloaded from the website of the Yinyin Yuan laboratory (http://www.yuanlab.org/). Samples were dichotomized about the median LI score into LI-low and LI-high groups. Lymphocytic lineages in the Immunological Genome Project were defined as all non-developmental B cells, T cells and NK cells. For each lymphocytic lineage, the mean CLS from the LI-high group was compared with that from the LI-low group using a two-sided *t*-test.

### Mutation count in TCGA data

Breast cancer (BRCA) level 3 somatic mutation data were accessed for breast cancer patients from the TCGA data portal (http://cancergenome.nih.gov/). Mutation count was calculated by summing all mutations that did not fall under the ‘silent' and ‘RNA' variant classification categories in the TCGA BRCA somatic mutation calling data. CLSs for each lineage were then calculated for each TCGA breast cancer sample and correlated with each sample's mutation count using the ‘cor' function in R. Lineages were then ranked by their Pearson's correlation coefficients. Relative enrichment at the top or bottom of the rankings for a given lineage subtype was calculated using a Wilcoxon rank-sum test that compared CLSs for lineages of that subtype with CLSs from lineages not of that subtype (background).

### Gene expression correlations

Gene expression levels for the CTLA-4 and TRAIL proteins were derived from the Curtis *et al.* data set under the gene symbols ‘*CTLA4*' and ‘*TNFSF10*', respectively. Expression values for each gene were correlated with CLSs for each haematopoietic lineage using the ‘cor' function in R. Lineages were then ranked based on their Pearson's correlation coefficients. Relative enrichment at the top or bottom of the rankings was calculated using the same procedure as for mutation count.

### Code availability

The R code for the BASE algorithm can be found in Supplementary Software 1.

## Additional information

**How to cite this article:** Varn, F. S. *et al.* Integrative analysis of breast cancer reveals prognostic haematopoietic activity and patient-specific immune response profiles. *Nat. Commun.* 7:10248 doi: 10.1038/ncomms10248 (2016).

## Supplementary Material

Supplementary Information Supplementary Figures 1-6.

Supplementary Data 1 Univariate Cox proportional hazard model results using haematopoietic CLSs in METABRIC data.

Supplementary Data 2 Significance of haematopoietic CLSs in METABRIC data in multiple Cox proportional hazards regression adjusting for age at diagnosis, stage, ER status, HER2 status, and PR status.

Supplementary Data 3 Gene signature matrix for Immunological Genome Project haematopoietic expression profiles.

Supplementary Data 4 Comparison of univariate Cox proportional hazards models for gene set CLSs vs. full haematopoietic expression profile CLSs in METABRIC data.

Supplementary Data5 Mean whole genome expression Pearson correlation coefficients for CD4+ naïve and Th cells vs developmental T cells.

Supplementary Data 6 Univariate Cox proportional hazards model results for METABRIC samples stratified by PAM50 subtype.

Supplementary Data 7 Univariate Cox proportional hazards model results for METABRIC samples stratified by Curtis integrative clustering subtypes.

Supplementary Data 8 Univariate Cox proportional hazards model results using CLSs across five breast cancer datasets.

Supplementary Data 9 Significant univariate Cox proportional hazards model results using haematopoietic CLSs in prostate cancer (GSE16560).

Supplementary Data 10 Significant univariate Cox proportional hazard model results using haematopoietic CLSs in non-small cell lung cancer (GSE8894).

Supplementary Data 11 Significant univariate Cox proportional hazards model results using haematopoietic CLSs in skin cutaneous melanoma (TCGA).

Supplementary Data 12 CLSs comparing murine haematopoietic profiles (Immunological Genome Project) with human haematopoietic profiles (Abbas et al).

Supplementary Data 13 CLSs comparing murine haematopoietic profiles (Immunological Genome Project) with human haematopoietic profiles (Novershtern et al).

Supplementary Data 14 Comparison of Cox proportional hazards model results between human haematopoietic profiles (Abbas et al) and their best-matched murine profiles (Immunological Genome Project).

Supplementary Software 1 BASE algorithm for use in R

## References

[b1] Junttila M. R. & de Sauvage F. J. Influence of tumour micro-environment heterogeneity on therapeutic response. Nature 501, 346–354 (2013). 24048067 10.1038/nature12626

[b2] Yoshihara K. *et al.* Inferring tumour purity and stromal and immune cell admixture from expression data. Nat. Commun. 4, 2612 (2013). 24113773 10.1038/ncomms3612 PMC3826632

[b3] Rooney M. S., Shukla S. A., Wu C. J., Getz G. & Hacohen N. Molecular and genetic properties of tumors associated with local immune cytolytic activity. Cell 160, 48–61 (2015). 25594174 10.1016/j.cell.2014.12.033 PMC4856474

[b4] Angelova M. *et al.* Characterization of the immunophenotypes and antigenomes of colorectal cancers reveals distinct tumor escape mechanisms and novel targets for immunotherapy. Genome Biol. 16, 64 (2015). 25853550 10.1186/s13059-015-0620-6 PMC4377852

[b5] Newman A. M. *et al.* Robust enumeration of cell subsets from tissue expression profiles. Nat. Methods 12, 453–457 (2015). 25822800 10.1038/nmeth.3337 PMC4739640

[b6] Jojic V. *et al.* Identification of transcriptional regulators in the mouse immune system. Nat. Immunol. 14, 633–643 (2013). 23624555 10.1038/ni.2587 PMC3690947

[b7] Shay T. *et al.* Conservation and divergence in the transcriptional programs of the human and mouse immune systems. Proc. Natl Acad. Sci. USA 110, 2946–2951 (2013). 23382184 10.1073/pnas.1222738110 PMC3581886

[b8] Cheng C., Yan X., Sun F. & Li L. M. Inferring activity changes of transcription factors by binding association with sorted expression profiles. BMC Bioinformatics 8, 452 (2007). 18021409 10.1186/1471-2105-8-452 PMC2194743

[b9] Curtis C. *et al.* The genomic and transcriptomic architecture of 2,000 breast tumours reveals novel subgroups. Nature 486, 346–352 (2012). 22522925 10.1038/nature10983 PMC3440846

[b10] Yuan Y. *et al.* Quantitative image analysis of cellular heterogeneity in breast tumors complements genomic profiling. Sci. Transl. Med. 4, 157ra143 (2012). 10.1126/scitranslmed.3004330 23100629

[b11] Ma X. J., Dahiya S., Richardson E., Erlander M. & Sgroi D. C. Gene expression profiling of the tumor microenvironment during breast cancer progression. Breast Cancer Res. 11, R7 (2009). 19187537 10.1186/bcr2222 PMC2687710

[b12] Man Y. G. *et al.* Tumor-infiltrating immune cells promoting tumor invasion and metastasis: existing theories. J. Cancer 4, 84–95 (2013). 23386907 10.7150/jca.5482 PMC3564249

[b13] Nakano O. *et al.* Proliferative activity of intratumoral CD8(+) T-lymphocytes as a prognostic factor in human renal cell carcinoma: clinicopathologic demonstration of antitumor immunity. Cancer Res. 61, 5132–5136 (2001). 11431351

[b14] Zhang L. *et al.* Intratumoral T cells, recurrence, and survival in epithelial ovarian cancer. N. Engl. J. Med. 348, 203–213 (2003). 12529460 10.1056/NEJMoa020177

[b15] Sato E. *et al.* Intraepithelial CD8+ tumor-infiltrating lymphocytes and a high CD8+/regulatory T cell ratio are associated with favorable prognosis in ovarian cancer. Proc. Natl Acad. Sci. USA 102, 18538–18543 (2005). 16344461 10.1073/pnas.0509182102 PMC1311741

[b16] Liakou C. I., Narayanan S., Ng Tang D., Logothetis C. J. & Sharma P. Focus on TILs: Prognostic significance of tumor infiltrating lymphocytes in human bladder cancer. Cancer Immun. 7, 10 (2007). 17591743 PMC2935746

[b17] Ohtani H. Focus on TILs: prognostic significance of tumor infiltrating lymphocytes in human colorectal cancer. Cancer Immun. 7, 4 (2007). 17311363 PMC2935759

[b18] Mahmoud S. M. *et al.* Tumor-infiltrating CD8+ lymphocytes predict clinical outcome in breast cancer. J. Clin. Oncol. 29, 1949–1955 (2011). 21483002 10.1200/JCO.2010.30.5037

[b19] Negrini S., Gorgoulis V. G. & Halazonetis T. D. Genomic instability--an evolving hallmark of cancer. Nat. Rev. Mol. Cell. Biol. 11, 220–228 (2010). 20177397 10.1038/nrm2858

[b20] Al-Hajj M., Wicha M. S., Benito-Hernandez A., Morrison S. J. & Clarke M. F. Prospective identification of tumorigenic breast cancer cells. Proc. Natl Acad. Sci. USA 100, 3983–3988 (2003). 12629218 10.1073/pnas.0530291100 PMC153034

[b21] Hanahan D. & Weinberg R. A. Hallmarks of cancer: the next generation. Cell 144, 646–674 (2011). 21376230 10.1016/j.cell.2011.02.013

[b22] Krummel M. F. & Allison J. P. CD28 and CTLA-4 have opposing effects on the response of T cells to stimulation. J. Exp. Med. 182, 459–465 (1995). 7543139 10.1084/jem.182.2.459 PMC2192127

[b23] Korman A. J., Peggs K. S. & Allison J. P. Checkpoint blockade in cancer immunotherapy. Adv. Immunol. 90, 297–339 (2006). 16730267 10.1016/S0065-2776(06)90008-X PMC1951510

[b24] Chan D. V. *et al.* Differential CTLA-4 expression in human CD4+ versus CD8+ T cells is associated with increased NFAT1 and inhibition of CD4+ proliferation. Genes Immun. 15, 25–32 (2014). 24173147 10.1038/gene.2013.57 PMC4284071

[b25] Fulda S. Tumor-necrosis-factor-related apoptosis-inducing ligand (TRAIL). Adv. Exp. Med. Biol. 818, 167–180 (2014). 25001536 10.1007/978-1-4471-6458-6_8

[b26] Cullen S. P. & Martin S. J. Fas and TRAIL ‘death receptors' as initiators of inflammation: Implications for cancer. Semin. Cell Dev. Biol. 39, 26–34 (2015). 25655947 10.1016/j.semcdb.2015.01.012

[b27] Falschlehner C., Schaefer U. & Walczak H. Following TRAIL's path in the immune system. Immunology 127, 145–154 (2009). 19476510 10.1111/j.1365-2567.2009.03058.x PMC2691779

[b28] Parker J. S. *et al.* Supervised risk predictor of breast cancer based on intrinsic subtypes. J. Clin. Oncol. 27, 1160–1167 (2009). 19204204 10.1200/JCO.2008.18.1370 PMC2667820

[b29] Chia S. K. *et al.* A 50-gene intrinsic subtype classifier for prognosis and prediction of benefit from adjuvant tamoxifen. Clin. Cancer Res. 18, 4465–4472 (2012). 22711706 10.1158/1078-0432.CCR-12-0286 PMC3743663

[b30] Ur-Rehman S., Gao Q., Mitsopoulos C. & Zvelebil M. ROCK: a resource for integrative breast cancer data analysis. Breast Cancer Res. Treat. 139, 907–921 (2013). 23756628 10.1007/s10549-013-2593-z

[b31] Wang Y. *et al.* Gene-expression profiles to predict distant metastasis of lymph-node-negative primary breast cancer. Lancet 365, 671–679 (2005). 15721472 10.1016/S0140-6736(05)17947-1

[b32] van de Vijver M. J. *et al.* A gene-expression signature as a predictor of survival in breast cancer. N. Engl. J. Med. 347, 1999–2009 (2002). 12490681 10.1056/NEJMoa021967

[b33] Schmidt M. *et al.* The humoral immune system has a key prognostic impact in node-negative breast cancer. Cancer Res. 68, 5405–5413 (2008). 18593943 10.1158/0008-5472.CAN-07-5206

[b34] Sboner A. *et al.* Molecular sampling of prostate cancer: a dilemma for predicting disease progression. BMC Med. Genomics 3, 8 (2010). 20233430 10.1186/1755-8794-3-8 PMC2855514

[b35] Lee E. S. *et al.* Prediction of recurrence-free survival in postoperative non-small cell lung cancer patients by using an integrated model of clinical information and gene expression. Clin. Cancer Res. 14, 7397–7404 (2008). 19010856 10.1158/1078-0432.CCR-07-4937

[b36] Abbas A. R. *et al.* Immune response in silico (IRIS): immune-specific genes identified from a compendium of microarray expression data. Genes Immun. 6, 319–331 (2005). 15789058 10.1038/sj.gene.6364173

[b37] Novershtern N. *et al.* Densely interconnected transcriptional circuits control cell states in human hematopoiesis. Cell 144, 296–309 (2011). 21241896 10.1016/j.cell.2011.01.004 PMC3049864

[b38] Kashii Y., Giorda R., Herberman R. B., Whiteside T. L. & Vujanovic N. L. Constitutive expression and role of the TNF family ligands in apoptotic killing of tumor cells by human NK cells. J. Immunol. 163, 5358–5366 (1999). 10553060

[b39] Kemp T. J., Elzey B. D. & Griffith T. S. Plasmacytoid dendritic cell-derived IFN-alpha induces TNF-related apoptosis-inducing ligand/Apo-2 L-mediated antitumor activity by human monocytes following CpG oligodeoxynucleotide stimulation. J. Immunol. 171, 212–218 (2003). 12817000 10.4049/jimmunol.171.1.212

[b40] Nelson B. H. CD20+ B cells: the other tumor-infiltrating lymphocytes. J. Immunol. 185, 4977–4982 (2010). 20962266 10.4049/jimmunol.1001323

[b41] Dunn G. P., Old L. J. & Schreiber R. D. The three Es of cancer immunoediting. Annu. Rev. Immunol. 22, 329–360 (2004). 15032581 10.1146/annurev.immunol.22.012703.104803

[b42] Klebanoff C. A., Gattinoni L. & Restifo N. P. CD8+ T-cell memory in tumor immunology and immunotherapy. Immunol. Rev. 211, 214–224 (2006). 16824130 10.1111/j.0105-2896.2006.00391.x PMC1501075

[b43] Webb J. R., Milne K., Watson P., Deleeuw R. J. & Nelson B. H. Tumor-infiltrating lymphocytes expressing the tissue resident memory marker CD103 are associated with increased survival in high-grade serous ovarian cancer. Clin. Cancer Res. 20, 434–444 (2014). 24190978 10.1158/1078-0432.CCR-13-1877

[b44] Djenidi F. *et al.* CD8+CD103+ tumor-infiltrating lymphocytes are tumor-specific tissue-resident memory T cells and a prognostic factor for survival in lung cancer patients. J. Immunol. 194, 3475–3486 (2015). 25725111 10.4049/jimmunol.1402711

[b45] Halaas O., Vik R., Ashkenazi A. & Espevik T. Lipopolysaccharide induces expression of APO2 ligand/TRAIL in human monocytes and macrophages. Scand. J. Immunol. 51, 244–250 (2000). 10736093 10.1046/j.1365-3083.2000.00671.x

[b46] Vivier E. *et al.* Innate or adaptive immunity? The example of natural killer cells. Science 331, 44–49 (2011). 21212348 10.1126/science.1198687 PMC3089969

[b47] Azimi F. *et al.* Tumor-infiltrating lymphocyte grade is an independent predictor of sentinel lymph node status and survival in patients with cutaneous melanoma. J. Clin. Oncol. 30, 2678–2683 (2012). 22711850 10.1200/JCO.2011.37.8539

[b48] Suzuki K. *et al.* Prognostic immune markers in non-small cell lung cancer. Clin. Cancer Res. 17, 5247–5256 (2011). 21659461 10.1158/1078-0432.CCR-10-2805

[b49] Gautier L., Cope L., Bolstad B. M. & Irizarry R. A. affy--analysis of Affymetrix GeneChip data at the probe level. Bioinformatics 20, 307–315 (2004). 14960456 10.1093/bioinformatics/btg405

