# Supplementary material for: Integrative analysis of breast cancer reveals prognostic haematopoietic activity and patient-specific immune response profiles
Source: Nat Commun. 2016 Jan 4;7:10248. doi: 10.1038/ncomms10248 (PMC4725766; doi:10.1038/ncomms10248)
Supplement: Supplementary Information — Supplementary Figures 1-6. [file ncomms10248-s1.pdf]

Supplementary Information

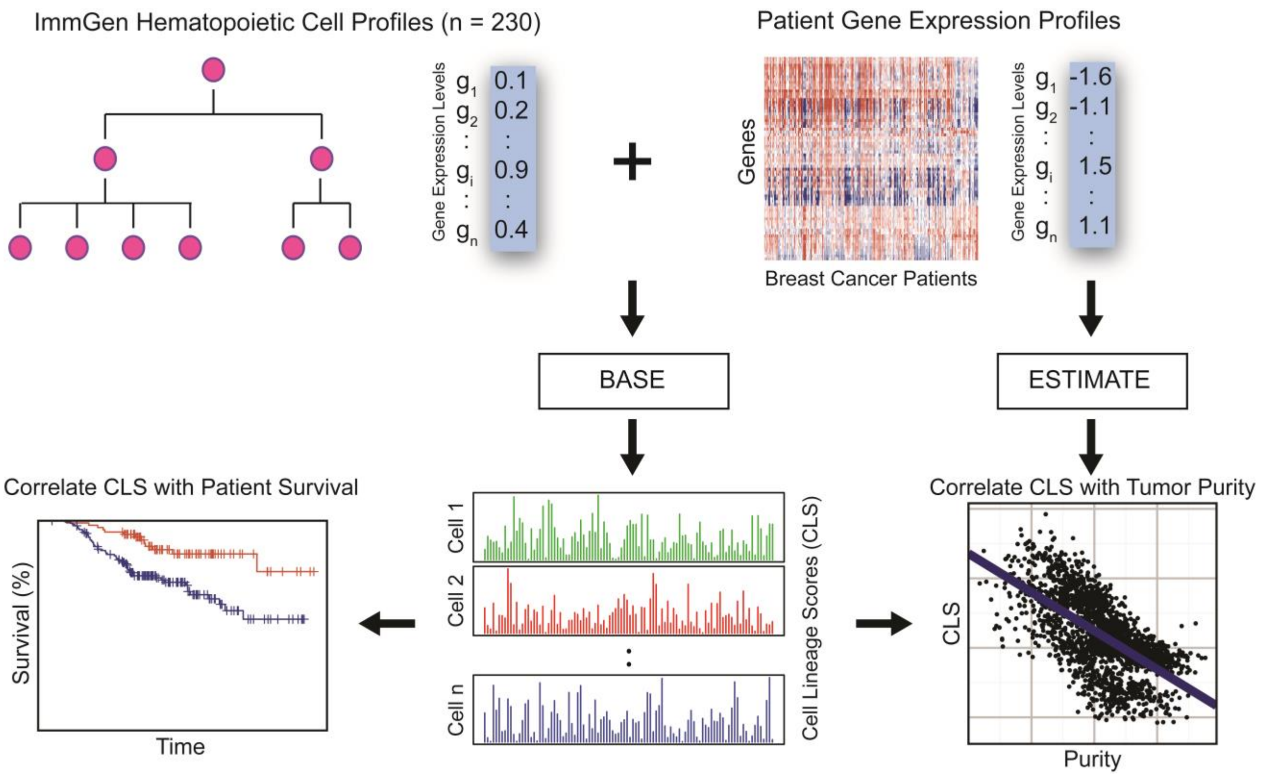

Supplementary Figure 1: Overview of workflow. Murine hematopoietic cell expression profiles were downloaded from the Immunological Genome Project and compared against gene expression profiles from a breast cancer dataset using the BASE algorithm. The resulting CLSs were then used as variables in a univariate Cox proportional hazards model to determine their association with patient survival. The CLSs were also correlated with tumor purity scores calculated by the ESTIMATE algorithm, which uses patient gene expression values to infer tumor purity. The resulting correlations indicated whether a CLS was a result of gene expression from tumor infiltrate, or indicative of a process intrinsic to the tumor itself.

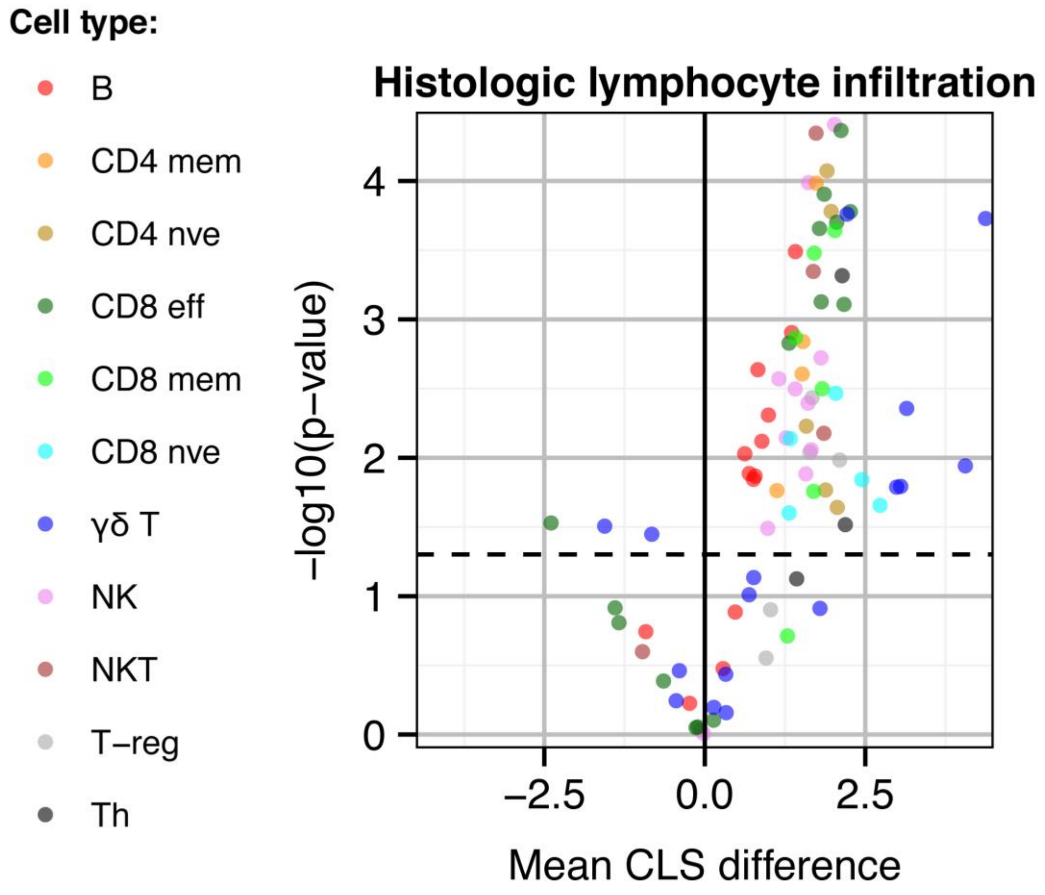

Supplementary Figure 2: Lymphocytic CLS differences between high and low LI groups. Distribution of CLS differences between LI-high and LI-low groups and the associated p-values (two-sided t-test). LI scores were calculated from eosin-stained tissue sections of 564 METABRIC samples. Samples were dichotomized about the median LI score into LI-high and LI-low groups. Lymphocytic lineages tested include B cells (B), CD4+ memory T cells (CD4 mem), CD4+ naïve T cells (CD4 nve), CD8+ effector T cells (CD8 eff), CD8+ memory T cells (CD8 mem), CD8+ naïve T cells (CD8 nve), γδ T cells (γδ T), natural killer cells (NK), natural killer T cells (NKT), regulatory T cells (T-reg), and T helper cells (Th).

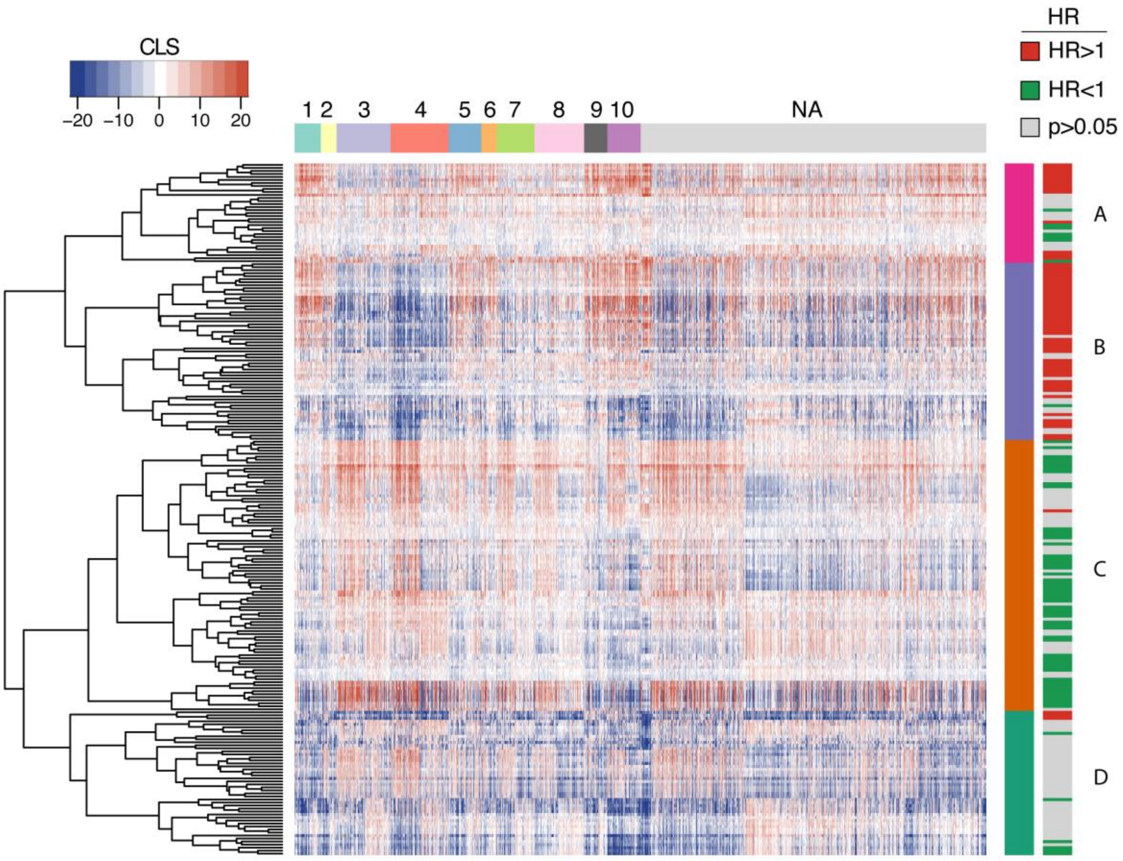

Supplementary Figure 3: Hematopoietic lineage association with integrative clustering subtypes. Heatmap depicting the hierarchical clustering of CLSs for each hematopoietic cell type in the integrative clustering subtypes by Curtis et al (n=76, 45, 156, 167, 94, 44, 109, 143, 67, 96, 995, for subtypes 1-10, and NA, respectively) (column sidebar). To show contrast, the max and min CLS were set to the 99th percentile of the original CLS distribution. Left row sidebar depicts the 4 distinct CLS clusters that emerged. Right row sidebar represents the hazard ratios for each hematopoietic cell type based on the univariate Cox proportional hazards survival analysis done in the Curtis dataset, with gray indicating adjusted p >0.05 (Wald test), green indicating HR >1, and red indicating HR <1.

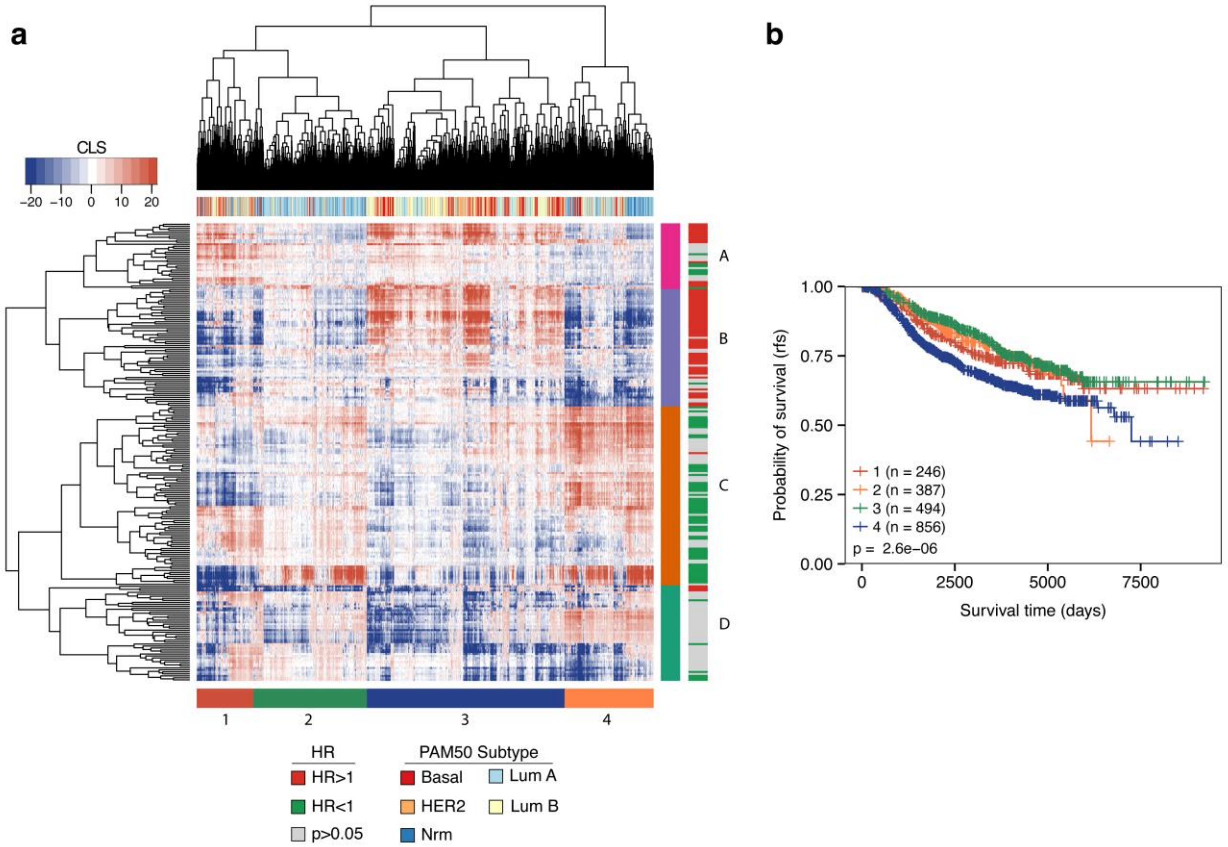

Supplementary Figure 4: Clustering of samples using hematopoietic CLS. (**a**) Heatmap depicting the hierarchical clustering of samples using CLSs for each hematopoietic cell type. To show contrast, the max and min CLS were set to the 99th percentile of the original CLS distribution. Column sidebar indicates the PAM50 subtype of each sample. Left row sidebar depicts the 4 distinct CLS clusters that emerged. Right row sidebar represents the hazard ratios for each hematopoietic cell type based on the univariate Cox proportional hazards survival analysis done in the Curtis dataset, with gray indicating adjusted $p > 0.05$ (Wald test), green indicating HR >1, and red indicating HR <1. (**b**) Kaplan-Meier plot depicting the survival probability over time for each cluster of samples defined in (a). Vertical hash marks indicate points of censored data. The four clusters had significantly different survival distributions ($p = 2.6e{-6}$, log-rank test).

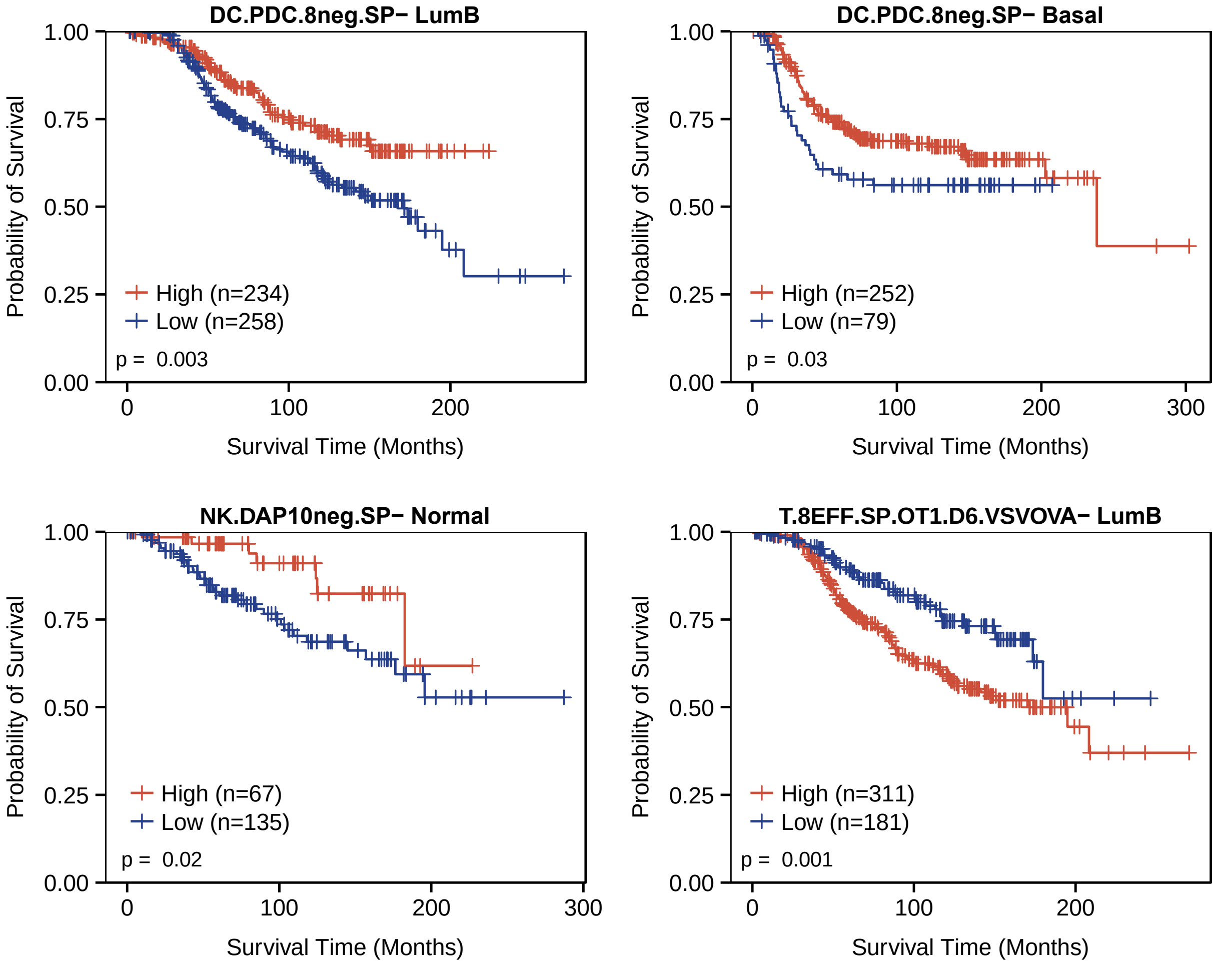

Supplementary Figure 5: Significant hematopoietic survival associations in PAM50 subtypes. Kaplan-Meier plots depicting the survival probability over time for samples of different PAM50 subtypes. Each plot stratifies samples into a high (red) and low (blue) hematopoietic CLS group for a different hematopoietic lineage. Samples were stratified into high and low groups based on whether their CLS was above or below the modal frequency of the subtype's CLS distribution for the given cell type. P-values were calculated using the log-rank test. Vertical hash marks indicate censored data.

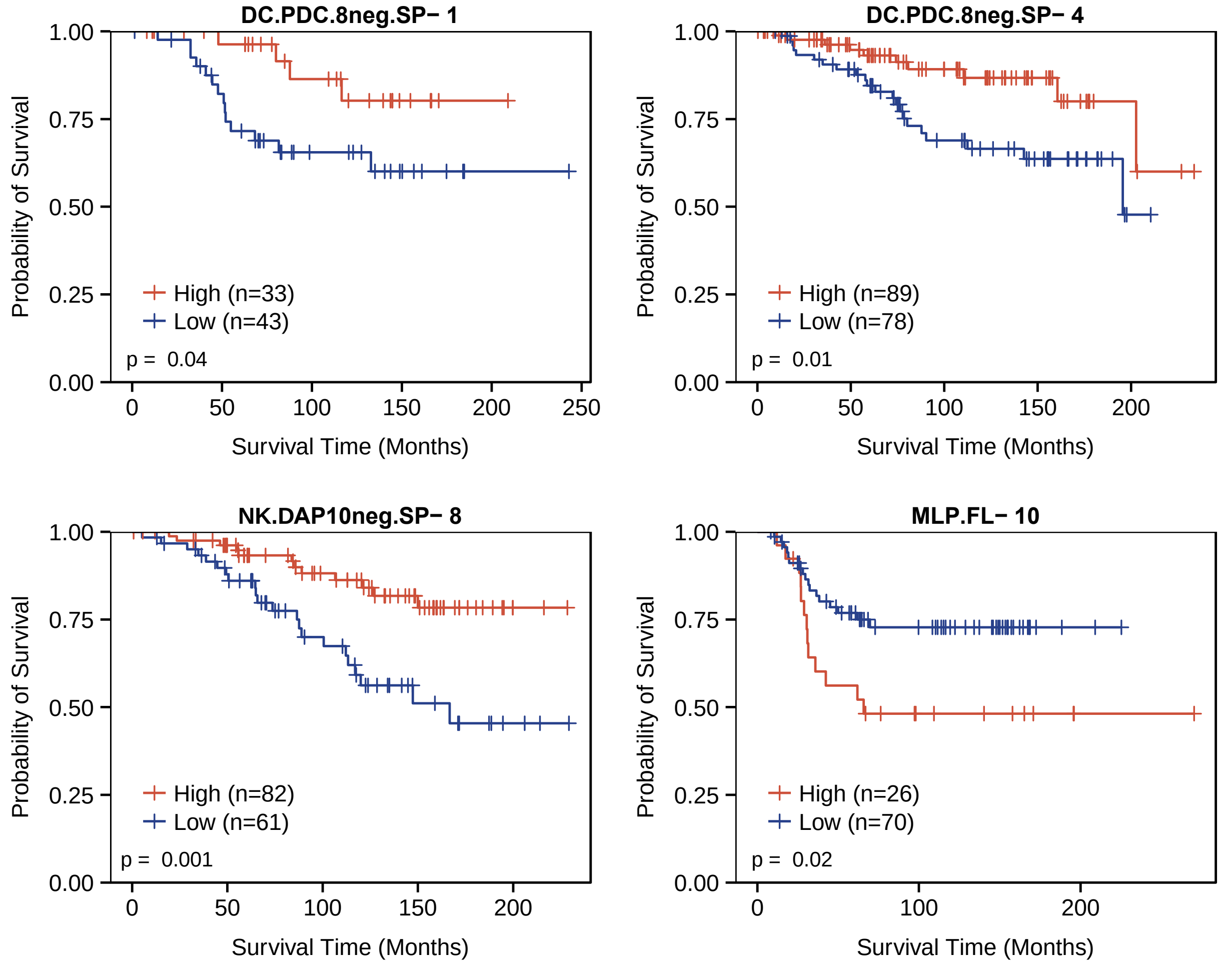

Supplementary Figure 6: Significant hematopoietic survival associations in integrative clustering subtypes. Kaplan-Meier plots depicting the survival probability over time for samples of the different integrative clustering subtypes by Curtis et al. Each plot stratifies samples into a high (red) and low (blue) hematopoietic CLS group for a different hematopoietic lineage. Samples were stratified into high and low groups based on whether their CLS was above or below the modal frequency of the subtype's CLS distribution for the given cell type. P-values were calculated using the log-rank test. Vertical hash marks indicate censored data.
