# Supplementary material for: Integrative analysis of breast cancer reveals prognostic haematopoietic activity and patient-specific immune response profiles
Source: Nat Commun. 2016 Jan 4;7:10248. doi: 10.1038/ncomms10248 (PMC4725766; doi:10.1038/ncomms10248)
Supplement: Supplementary Software 1 — BASE algorithm for use in R [file ncomms10248-s16.docx]

Supplementary Software 1: BASE algorithm for use in R

base <- function(data, reg, perm=100, myoutf, median.norm=T)

{

## quantile normalization

myrk = matrix(0, nrow(data), ncol(data))

xx = myrk

for(k in 1:ncol(data))

{

myrk[,k] = rank(data[,k])

xx[,k] = sort(data[,k])

}

mymed = apply(xx, 1, median, na.rm=T)

for(k in 1:ncol(data))

{

data[,k] = mymed[myrk[,k]]

}

comGene = intersect(row.names(data), row.names(reg))

data = data[comGene, ]

reg = reg[comGene, ]

if(median.norm)

{

mymed = apply(data, 1, median)

data = data-mymed

}

cnum = ncol(data)

rnum = nrow(data)

es = matrix(0, cnum, ncol(reg))

cat("\ncalculate ES\n")

for(k in 1:cnum)

{

cat("\r",k)

myorder = order(data[,k], decreasing=T)

cur.exp = data[myorder,k]

cur.reg = reg[myorder,]

fg1 = as.matrix(abs(cur.reg*cur.exp))

bg1 = as.matrix(abs((1-cur.reg)*cur.exp))

for(i in 2:nrow(fg1))

{

fg1[i,] = fg1[i,]+fg1[i-1,]

bg1[i,] = bg1[i,]+bg1[i-1,]

}

for(i in 1:ncol(fg1))

{

fg1[,i] = fg1[,i]/fg1[rnum,i]

bg1[,i] = bg1[,i]/bg1[rnum,i]

}

xx = fg1-bg1

tmp = apply(xx, 2, max)

pos.es = ifelse(tmp>0, tmp, 0)

tmp = apply(xx, 2, min)

neg.es = ifelse(tmp<0, tmp, 0)

es[k,] = ifelse(pos.es>abs(neg.es), pos.es, neg.es)

}

## perm

cat("\n Permutation \n")

cur.reg = reg

pos.es = neg.es = matrix(0, ncol(reg), perm)

for(k in 1:perm)

{

cat("\r", k)

se = sample(1:cnum, 1)

cur.exp = sample(data[,se])

fg1 = as.matrix(abs(cur.reg*cur.exp))

bg1 = as.matrix(abs((1-cur.reg)*cur.exp))

for(i in 2:nrow(fg1))

{

fg1[i,] = fg1[i,]+fg1[i-1,]

bg1[i,] = bg1[i,]+bg1[i-1,]

}

for(i in 1:ncol(fg1))

{

fg1[,i] = fg1[,i]/fg1[rnum,i]

bg1[,i] = bg1[,i]/bg1[rnum,i]

}

xx = fg1-bg1

tmp = apply(xx, 2, max)

pos.es[,k] = ifelse(tmp>0, tmp, 0)

tmp = apply(xx, 2, min)

neg.es[,k] = ifelse(tmp<0, tmp, 0)

}

## normalize

pavg = apply(pos.es, 1, mean)

navg = abs(apply(neg.es, 1, mean))

pos.npes = pos.es/pavg

neg.npes = neg.es/navg

for(k in 1:nrow(es))

{

tmp = es[k,]

es[k,] = ifelse(tmp>0, tmp/pavg, tmp/navg)

}

res = es

colnames(res) = paste(colnames(reg), ".ES", sep="")

row.names(res) = colnames(data)

write.table(res, myoutf, sep="\t", row.names=T, quote=F)

myList= NULL

myList[[1]] = es

names(myList) = "nes"

return(myList)

}
